# Gender Differences in Sinonasal Cancer Incidence: Data from the Italian Registry

**DOI:** 10.3390/cancers16112053

**Published:** 2024-05-29

**Authors:** Alessandra Binazzi, Davide di Marzio, Carolina Mensi, Dario Consonni, Lucia Miligi, Sara Piro, Jana Zajacovà, Denise Sorasio, Paolo Galli, Angela Camagni, Roberto Calisti, Stefania Massacesi, Ilaria Cozzi, Anna Balestri, Stefano Murano, Ugo Fedeli, Vera Comiati, Silvia Eccher, Sara Lattanzio, Alessandro Marinaccio, ReNaTuNS Working Group

**Affiliations:** 1 Department of Occupational and Environmental Medicine, Epidemiology, Hygiene, National Institute for Insurance against Accidents at Work (INAIL), 00100 Roma, Italy; d.dimarzio@inail.it (D.d.M.); a.marinaccio@inail.it (A.M.); 2 Sinonasal Cancer Registry of Lombardy, Epidemiology Unit, Fondazione IRCCS Ca’ Granda Ospedale Maggiore Policlinico, 20100 Milano, Italy; carolina.mensi@unimi.it (C.M.); dario.consonni@unimi.it (D.C.); 3 Institute for Cancer Research, Prevention and Clinical Network (ISPRO) Foundation, 50139 Firenze, Italy; l.miligi@ispro.toscana.it; 4 Sinonasal Cancer Registry of Tuscany, Occupational and Environmental Epidemiology Unit, (ISPRO), 50139 Firenze, Italy; s.piro@ispro.toscana.it; 5 Sinonasal Cancer Registry of Piedmont, Occupational Health and Safety Department, CN1 Local Health Authority, 12037 Saluzzo, Italy; jana.zajacova@aslcn1.it (J.Z.); denise.sorasio@aslcn1.it (D.S.); 6 Sinonasal Cancer Registry of Emilia Romagna, Occupational Safety and Prevention Unit, Public Health Department, Bologna Local Health Authority, 40121 Bologna, Italy; paolo.galli@ausl.bologna.it (P.G.); angela.camagni@ausl.bologna.it (A.C.); 7 Sinonasal Cancer Registry of Marche, Unit of Workplace Prevention and Safety and of Occupational Epidemiology (SPreSAL Epi Occ), Department of Prevention, Macerata Health Authority, 62012 Civitanova Marche, Italy; roberto.calisti@sanita.marche.it (R.C.); stefania.massacesi@sanita.marche.it (S.M.); 8 Sinonasal Cancer Registry of Lazio, Department of Epidemiology, Lazio Regional Health Service, ASL Roma 1, 00147 Roma, Italy; i.cozzi@deplazio.it (I.C.); anna.balestri@asl.vt.it (A.B.); 9 Laboratory of Industrial Hygiene, Department of Prevention, CRRA ASL Lazio, 01100 Viterbo, Italy; 10 Sinonasal Cancer Registry of Autonomous Province of Bolzano, Alto Adige Health Authority, Occupational Medicine Unit, 39100 Bolzano, Italy; stefano.murano@sabes.it; 11 Azienda Zero, Epidemiological Department, Veneto Region, 35131 Padova, Italy; ugo.fedeli@azero.veneto.it (U.F.); vera.comiati@azero.veneto.it (V.C.); 12 Sinonasal Cancer Registry of Autonomous Province of Trento, Hygiene and Occupational Medicine, Provincial Unit of Health, 38123 Trento, Italy; silvia.eccher@apss.tn.it (S.E.); sara.lattanzio@apss.tn.it (S.L.)

**Keywords:** sinonasal cancer, occupational exposure, gender difference, epidemiological surveillance

## Abstract

**Simple Summary:**

Sinonasal cancer (SNC) is strongly associated with occupational exposure to several carcinogens involved in SNC’s etiology, which vary by gender. Gender differences in SNC cases were examined through the Italian sinonasal cancer registry. Male-to-female incidence differences are neglectable in the youngest age classes but increase in older age classes, probably as a result of more men being diagnosed with SNC due to their greater occupational exposure to carcinogens (mostly wood and leather dusts) compared with women. Occupational exposures to carcinogens were the most frequent in both genders. A high percentage of women had unlikely exposures. Gender differences deserve more deep investigation, starting with a review of diagnostic processes and occupational history taking.

**Abstract:**

Background: Although rare, sinonasal cancers (SNCs) have a high occupational attributable fraction. Methods: We applied gender-based approaches to descriptive analyses, incidence, and patterns of exposures using the Italian National Sinonasal Cancer Registry (ReNaTuNS: Registro Nazionale Tumori Naso-Sinusali). Results: The study included 2851 SNC patients. SNC was diagnosed more often in men (73%) than in women (27%). The most frequent morphology in men was intestinal-type adenocarcinoma (33%), whereas in women, it was squamous cell carcinoma (49%). Nasal cavities were predominant in both genders (50%), ethmoidal sinus in men (24%), and maxillary in women (24%). Incidence rates were 0.76 (per 100,000 person-years) in men and 0.24 in women and increased by age, more evidently in men, peaking over 75 years in both. Occupational exposures to wood and leather dusts were the most frequent (41% for men, 33% for women). Few exposures were extra-occupational or domestic. Unlikely exposure was relevant in women (57%). Conclusions: The surveillance of SNC cases through a registry that allows for the identification of and compensation for this occupational disease is important in Italy, where numerous workers are exposed to carcinogens for SNC, without even being aware. Considering the rarity of the disease, particularly among women, the ReNaTuNS can provide a method to analyze gender differences.

## 1. Introduction

Sinonasal cancer (SNC) includes cancers of the nasal cavity and paranasal sinuses and represents the cancer site with the second-highest occupational attributable fraction (AF) after mesothelioma induced by asbestos. The relationship between specific occupational exposures (in particular, wood and leather dusts) and the development of SNC has been well documented [1]. In the United Kingdom, the occupational AF for SNC was estimated at 43% for men and 20% for women in 2004 [2]. Recently, a study concluded that 12% of SNCs could be attributed to occupational exposures. The authors exclusively focused on the causative role of wood dusts, omitting other carcinogens for SNC [3]. SNC has also been associated with Human Papilloma Virus (HPV), mostly the squamous cell carcinoma subtype [4,5,6].

SNCs are rare diseases and constitute only less than 1% of all cancers and 3% to 5% of cancers in the upper respiratory tract, with an overall incidence estimated at 0.5–1 case per 100,000 [7]. Squamous cell carcinoma (SCC) and adenocarcinoma (AD) are the most common histologic subtypes, together accounting for almost 80% of all sinonasal tumors.

Almost half of cases occur in the nasal cavities, around one-third in the maxillary sinuses, and one-tenth in the ethmoidal sinuses. Less than 3% originate in the frontal and sphenoidal sinuses [8].

Overall outcomes of sinonasal cancers (SNCs) are poor, with 5-, 10-, and 20-year survival estimated at 46%, 32%, and 16%, respectively, depending on specific histologic subtype [9]. Because of the anatomic location, SNCs often have an unspecific onset of symptoms, and patients often present with advanced-stage tumors; therefore, they have a poor prognosis. Moreover, male sex and older age at diagnosis were associated with reduced survival [10]. A recently published Italian study observed that age and cancer site had time-dependent effects on prognosis; especially within the first month after diagnosis, cancer morphology significantly influenced the prognosis, without differences between women and men [11].

However, the clinical management of SNC has recently benefited from advancements in imaging techniques, endoscopic surgical approaches, and radiotherapy [12].

SNCs are reported to be 1.8 times more frequent in males than females and in older adults, with 80% diagnosed at 55 years of age or older [9,13].

There have been limited studies exploring geographic variations in the epidemiology of SNCs in different regions of the world. A population-based comparison of European and North American sinonasal cancer survival found that SNC in Europe and the US most commonly affects males and individuals over the age of 55 years. Male gender and age over 75 are poor prognostic factors at 5 years. Five-year relative survival in the US is higher than in Europe [14].

Prevalence of head and neck cancers differs between males and females, with a higher probability to become ill for males. This may imply sex-specific biological differences or environmental situations that differently affect males and females [15].

In this study, we investigate gender differences in SNC by relying on the Italian National Sinonasal Cancer Registry (“ReNaTuNS”: Registro Nazionale dei Tumori Naso-Sinusali), established at the Italian Workers’ Compensation Authority (INAIL: Istituto Nazionale per l’Assicurazione contro gli Infortuni sul Lavoro).

## 2. Materials and Methods

The ReNaTuNS is based on Regional Operational Centers (“COR”: Centri Operativi Regionali) that acquire clinical records from departments of SNC diagnosis and treatment. Primary malignant epithelial cancers of the nasal cavity and paranasal sinuses are selected. Histological characterization of epithelial SNC cases follows the WHO 2017 classification of head and neck cancers [16].

Standardized questionnaires, administered to patients or their next of kin by trained interviewers, evaluate the exposure history. Exposure to known/suspected carcinogens is evaluated by a panel of expert industrial hygienists. Cases are allocated to distinct exposure categories using a standardized reference grid, which primarily considers carcinogenic agents with either sufficient or limited evidence for SNCs in humans listed by IARC [1], and thereafter other agents suggested by epidemiological studies.

Following such a grid, the occupational exposure is given to individuals with a history of employment implying exposure to the causal agent in their workplace. Coding of occupational histories is based on Italian classification of industries and occupations [17]. Multiple exposures occur when subjects have exposures attributed to various economic sectors or jobs or due to diverse causal carcinogenic agents. Exposure may differ depending on the likelihood of exposure (certain, probable, or possible) during employment history. Non-occupational exposure pertains to individuals exposed in non-work-related settings (environmental, domestic, or during leisure activities). An unlikely exposure concerns subjects with available information about working activities and lifestyle, from which an exposure to the causal agent can either be ruled out or cannot be identified. Finally, the maximum level of exposure registered across multiple periods is assigned to each subject, and mutually exclusive exposure categories are classified in the following descending hierarchical order: occupational, domestic, leisure activities, unlikely. All the procedures mentioned above are detailed in the national guidelines for the keeping of the registry that ensure data quality [18].

Gender differences were investigated with regard to age at diagnosis, histologic type, SNC subsite, incidence, and exposure to carcinogens using the ReNaTuNS database.

Reporting SNC cases to the ReNaTuNS is compulsory by Italian law (Italian Legislative Decree n. 81/2008). Therefore, ethics approval by an ethics committee or Institutional Board is not required. Participants gave informed consent to participate in the study before taking part.

Descriptive statistics were used to record baseline characteristics. Males and females were compared by means of the χ^2^ test. Gender-specific, age-standardized incidence rates per 100,000 were computed, using the 2013 European standard population as a reference. Incidence rate ratios (IRR) with 95% confidence intervals (95% CI) were calculated to compare male-to-female incidence. A significance level of 0.05 was used for all statistical analyses. Data were analyzed using IBM SPSS, version 25.0 (IBMSPSS, Armonk, New York, NY, USA).

## 3. Results

The present study included 2851 patients affected by epithelial SNC, reported to the ReNaTuNS and with incidence between 1989 and 2022 (Table 1). Within the study population, 73% (N = 2073) occurred in males and 27% (N = 778) in females, resulting in a male-to-female ratio of 2.6. The mean age at diagnosis was 67 years for males and 66 for females (the median was 68 for both). There was a higher percentage of women below the age of 55 (22% compared with 16% in men) (*p* < 0.05). Half of all SNC tumors originated from the nasal cavity in both genders. A difference was observed in maxillary and ethmoidal sinuses, the former being more represented in females (24%) and the latter in males (24%) (*p* < 0.0001). Smaller percentages were found for the other anatomical sites. By histology, intestinal-type adenocarcinomas (ITACs) were more frequent in males (33%) than females (10%), whereas squamous cell carcinomas (SCCs) were more frequent in females (49%) than males (36%) (*p* < 0.0001). Salivary gland carcinomas (SGCs) were also more common in females (14%) (Table 1). ITACs were strongly associated with the ethmoidal sinus in both genders (*p* < 0.001).

The overall age-standardized SNC incidence rates (cases per 100,000) calculated from 2015 to 2018 (period in which the incidence was complete in the Italian Regions included in the registry, mapped in Figure S1) were 0.76 in males and 0.24 for females.

Higher incidence rates in men than in women were observed across all age classes, cancer sites, and main histologic groups. Within these strata, the male-to-female incidence rate ratio (IRR) had the lowest value for the 25–34 age category (IRR: 0.98; 95% CI: 0.28–3.37) and the highest for adenocarcinoma (IRR: 5.00; 95% CI: 3.83–6.53) (Table 2).

**Figure 1:**
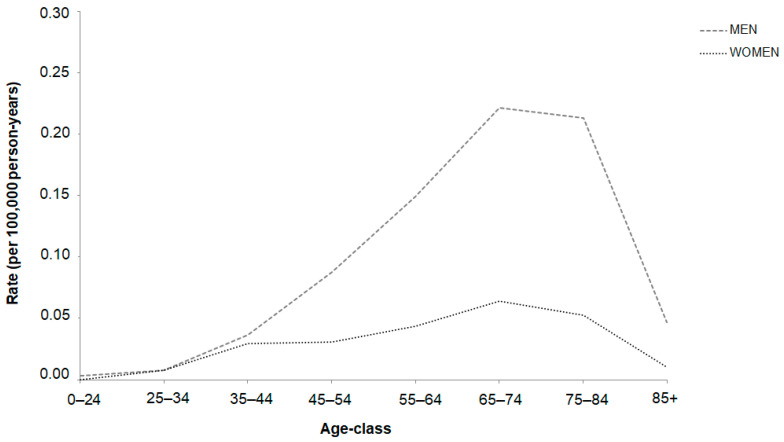
Trend of SNC incidence by age-specific rates (per 100,000 person-years) by gender (period: 2015–2018).

The incidence rates of SNC showed an increase with age, with a more pronounced rise in males. The highest rates were observed among patients in the age group of 75–84 years, followed by a gradual decline in rates with advancing age. Interestingly, the male-to-female ratio exhibited an upward trend, starting from the 25–34 age group, with a steep ascent (Figure 1).

The overall male-to-female IRR was 3.10 (95% CI: 2.67–3.61), but it varied across the Italian Regions (Figure S2).

**Figure 2:**
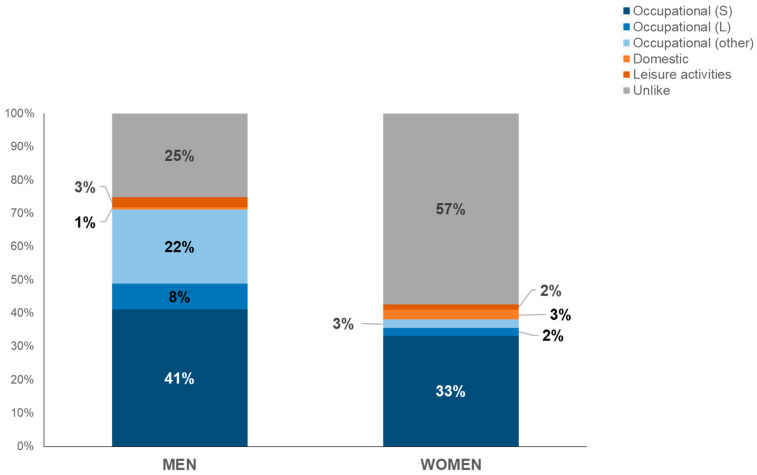
Distribution of types of exposure by gender (period: 1989–2022). Note: S = carcinogenic agents with sufficient evidence in humans for SNCs; L = carcinogenic agents with limited evidence in humans for SNCs.

Information on occupational and non-occupational exposures was available for 76.3% of SNC cases (1635 men and 540 women); most were occupational exposures to the carcinogenic agents with sufficient evidence in humans for SNCs (41% for men and 33% for women). Wood dust involved 43% of male and 19% of female exposures, and leather dust involved, respectively, 25% and 30%. Regarding carcinogenic agents with limited evidence in humans for SNCs, exposures were found to hexavalent chromium compounds (4% in men, 3% in women), formaldehyde (2.6% in men, 6.2% in women), and textile dusts (1% in men, 14% in women). Furthermore, a small number of exposures to other agents, suggested by the epidemiological literature, concerned solvents (10% in men, 14% in women) and—in a range declining from 10% to 1% in both genders—pesticides, polycyclic aromatic hydrocarbons (PAHs), flours, and silica. Few exposures were extra-occupational or domestic. The percentage of unlikely exposure was particularly relevant in women (57%) (Figure 2).

The median year of first exposure for subjects with an occupational exposure to the carcinogenic agents with sufficient evidence in humans for SNCs was 1959 for males (range: 1927–2014) and 1964 for females (range: 1930–2011). Mean latency—calculated as the difference between incidence year and onset of exposure—was 50.1 (SD: ±13.3) for males and 46.6 (SD: ±14.6) for females.

When considering the type of exposure detection through interviews, most of the information was directly obtained from the patient (61% males, 51% females), and a minority was obtained indirectly, i.e., from a next of kin (16% males and 19% females). The percentages of subjects without an interview were 22% in males and 29% in females, and 1% of cases were not tracked for an interview. Regarding the distribution of exposures by type of interview, the percentage of cases with occupational exposure was higher when the interview was direct. Such differences were more evident in males than in females (Figure S3).

**Figure 3:**
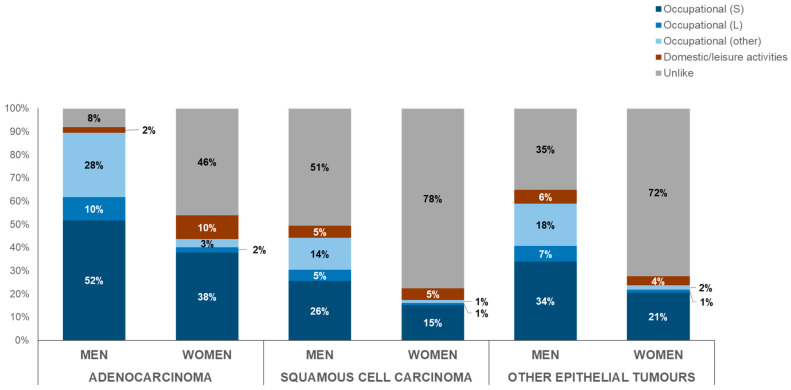
Distribution of type of exposure by morphology and gender (period: 1989–2022). Note: S = carcinogenic agents with sufficient evidence in humans for SNCs; L = carcinogenic agents with limited evidence in humans for SNCs.

The majority of interviewed subjects diagnosed with AD had an occupational exposure (males: 90%; females: 44%), but a considerable number of cases with SCC (males: 44%; females: 17%) and with other epithelial tumors (males: 59%; females: 24%) also had occupational exposures (Figure 3).

For patients occupationally exposed to wood and leather dusts, a strong association between ITAC and ethmoidal sinus was confirmed (*p* < 0.001). Disentangling by gender, the association persisted only in men (*p* < 0.05).

Smoking data were available for 81% of female (37% current smokers, 22% former smokers, 41% non-smokers) and 89% of male respondents (43% current smokers, 40% former smokers, 17% non-smokers).

## 4. Discussion

Gender differences in cancer incidence and mortality may be the result of a complex interaction between environmental and genetic factors. Moreover, socioeconomic inequalities can affect cancer occurrence differently in men and women, by involving unhealthy lifestyle behaviors, smoking habits, alcohol consumption, awareness of risks, occupational exposure, and access to healthcare [19]. Overall, the probability of developing cancer is greater in men than in women [20].

Head and neck cancers are characterized by a higher incidence in males than in females. In males, apart from factors such as alcohol and smoking, a possible role of sex hormones has been described. Some clinical studies on head and neck cancer patients have shown that estrogens levels in females may play a protective role in developing cancer [21].

In the United States, a study described a male-to-female cancer incidence ratio of 2.63 for cancers of the oral cavity, 4.32 for the pharynx, and 3.96 for the larynx [22]. A Korean study found a 1.8-fold higher risk for SNC in males than in females [15]. In the present study, the overall IRR for SNC was 3.10, but it varied throughout the regions, probably reflecting the local industrial contexts, with a prevalence of male workers in several sectors (woodworking and others). Moreover, within some individual activities, the prevalence of male or female workers occurs in specifically dangerous tasks (e.g., leather sole trimming in footwear manufacturing in males and upper construction and finishing, involving the use of adhesives and solvents and, therefore, exposure to solvent vapors, in females).

In Italy, the registration of SNC cases covers a geographical area of nearly 220,000 km^2^ (73% of the national territory) and more than 51 million inhabitants (87% of the national population). Age-standardized incidence rates per 100,000 person-years were higher in men than in women (0.76 vs. 0.24), a difference that was also found by the Italian Cancer Registries Association (0.67 in men and 0.34 in women) [23] and in a study in Taiwan (2.3 vs. 1.0) [24]. Furthermore, a higher proportion of male cases (63%) was observed in a Danish study [25]. Data from 94 European cancer registries (2000–2007) reported an estimate of 0.45 per 100,000 [26].

The gap of incidence rates over time between men and women is narrower until the age of 44 (possibly due to the rarity of disease in young people), then widens and rises in both genders, especially in males. Although the present study included more men than women with SNC, the proportion of younger women was greater than that of younger men (22% vs. 16% under 55 years old).

Gender gaps were confirmed when also stratifying by tumor subsites and histology. Except for the nasal cavity, which involved half of the tumors in both genders, differences concerned maxillary sinus (more represented in females) and ethmoidal sinus (in males).

The proportion by localization was similar to other studies [8,27,28,29]. In the present study, about 40% of histological types were SCC, which is the most common type (more than 50% described in the literature) and affects male subjects more frequently, with a male-to-female ratio of 2:1 [30]. SCC risk has been associated with exposure to arsenic and welding fumes [31], and emerging data address a possible role of HPV [32,33]. In this regard, the ReNaTuNS questionnaire does not ask the patient about a previous HPV infection, nor is the information registered in the database if the pathologist tests for immunohistochemical positivity.

ADs represent about 20% of the malignant neoplasms of the sinonasal tract and affect male subjects more frequently: our results of a 6.6 male-to-female ratio are consistent with the value up to 6.1 reported in the literature [34]. High incidence of AD has been associated with occupational exposure to wood dust, possibly following a pathway driven by persistent inflammation that leads to tumor formation [12,35] that is more frequent among men and may reasonably explain the male prevalence of AD [36]. Among AD, the ITAC subtype seems to be more frequently associated with wood dust than the non-ITAC subtype [37]. The results of our study confirm the correlation between ITACs and ethmoid sinus for occupational exposure to both wood and leather dusts in men. A high proportion of ITAC (87% of the enrolled cases) with occupational exposure to leather or hardwood dusts was shown by an Italian study [38]. However, a geographical variation exists in ITAC patients [39], which may be attributed to the presence of unidentified genetic susceptibility factors or exposure to different types of wood or other compounds.

In any case, AD is not a unique histologic type involved in occupational exposure, as reported by some studies [40]; non-negligible proportions for SCC and other epithelial SNCs as well were found in this study.

Smoking is an important risk factor for SNC, particularly for SCC in current smokers [30,35,41]. A 60% increase in risk was found in ever-smokers (95% CI: 24–107%), along with an increase of 6% per pack-year (95% CI: 5–7%) smoked, with the risk significantly decreasing after quitting smoking [42]. Moreover, smoking is correlated with worse outcomes in SCCs, with current smokers having a decreased 5-year overall survival compared with former smokers [43]. Smoking patterns differ between genders, with a higher prevalence in men, although in recent years, women’s smoking has increased, as result of changing social and environmental determinants. The ReNaTuNS standardized questionnaire includes items related to smoking, and a high rate of response was obtained (although answering is not mandatory), with a prevalence of current and former smokers among men. However, this study focused exclusively on SNC patients; therefore, we cannot devise any statistical inference to investigate the relationship between smoking and SNC. This is a subject to address in potential future research, e.g., through an analytical design like a case-control study involving the ReNaTuNS cases recorded.

No role for alcohol intake as a risk factor for SNC has been recognized [44], although—together with tobacco smoking—it is considered one of the major factors responsible for the incidence of head and neck cancers [13].

The study’s strengths lean on the use of a comprehensive registration of cases through a dedicated cancer registry. Through a standardized questionnaire, the exposure to occupational carcinogens is assessed for a significant portion of patients, and findings are supported by other studies [31,45,46]. Collecting the lifetime work history from each SNC case allows for recognizing occupational diseases and eventually compensating the affected subjects.

Occupational exposures contribute greatly to SNC incidence. The observed higher incidence in males and the distinct trends in incidence between males and females may be indicative of previous different exposures to risk factors [29]. In this study, 41% of male and 33% of female exposures occurred in workplaces, mainly due to wood and leather dusts (the former was more represented in men, the latter in women).

The advanced mean age at diagnosis and the widening gender gap in the older population suggest that occupational exposures are more easily recognized in men, possibly owing to more awareness among healthcare professionals of SNC risk in male-specific work settings. In women, the scarcity of available information could imply an underestimate of occupational risk factors. Occupational differences between men and women with SNC warrant more insight in obtaining occupational histories, with stronger training required from interviewers. In our study, women’s exposure history was more often classified as unlikely with respect to men, and this could include indirect exposure in the work environment.

The weakness of this study is the lack of homogeneous SNC registration in the Italian Regions, although there have been recent improvements thanks to an update of the national guidelines [18] and to a dedicated project for strengthening the CORs network.

Moreover, due to their anatomic location and the late-stage presentation with unspecific onset of symptoms, sinonasal malignancies are challenging to diagnose early. Sources of possible non-differential misclassification affect the ability of identifying the site of first occurrence correctly, mainly for the advanced cases, in particular for the vestibular squamous-cell type neoplasms (whether arising from the skin or from the nasal cavity), the neoplasms involving the maxillary sinus (whether arising from the oral mucosa or from the sinonasal mucosa), and the neoplasms involving both the upper nasal cavities and the nasopharynx (if arising from one or the other of the two sites). The ReNaTuNS classifies the irresolvable situations as “probable” cases [18].

Finally, obtaining information from patients, generally of advanced age, with associated noteworthy morbidity and aesthetic complications, is difficult. The reason for the higher proportion of missed interviews among women compared to men may be due to this specific issue. Furthermore, in non-interviewed subjects, we have no data about occupational history, and this remains a study limit.

Occupational epidemiology has frequently disregarded female workers, mainly because the category had a low level of representativeness in the past workforce or was segregated in specific industrial sectors. In fact, the presence of women is relevant in specific sectors (e.g., the textile and shoe industries, healthcare, teaching), where gender differences in occupational risk factors may become apparent [47].

Therefore, intensifying studies about women is essential, mainly in those sectors with greater employment of female workers, where the exposure to carcinogens is recognized. It is recommended to deeply investigate the exposure history from a gender perspective and to arrange epidemiological studies on occupational cancer in the female workforce, to manage health promotion and preventive dedicated efforts in the workplaces, and to assure welfare compensations in the case of onset of occupational diseases.

## 5. Conclusions

This study shows how a national population-based registry with a complete collection of SNC cases allows for a comparison of male-to-female incidence, site of SNC origin, morphology, and patterns of exposure. Given both the rarity of disease, especially in women, and the strong occupational etiology, the ReNaTuNS can offer a way to investigate gender differences in occupational studies. This is relevant in the globally growing evidence regarding the impact of gender differences on cancer susceptibility, development, survival, treatment response, and prevention. This variability implies a better understanding of complex factors and their interactions and deserves more awareness in research that is equally representative of women and men.

## Figures and Tables

**Table 1:** Characteristics of SNC cases registered in the ReNaTuNS database (period: 1989–2022).

Variable	Men N (%)	Women N (%)
Sex	2073 (73)	778 (27)
Age, years (mean ± SD)	67 ± 12.4	66.0 ± 15.2
Age class		
0–44	104 (5)	82 (11)
45–54	230 (11)	92 (12)
0–54	334 (16)	174 (22)
55–64	460 (22)	142 (18)
65–74	639 (31)	195 (25)
75+	640 (31)	267 (34)
55+	1739 (84)	604 (78)
Topography (ICD-X codes)		
Nasal cavities (C30.0)	1204 (50)	452 (50)
Maxillary sinus (C31.0)	420 (17)	220 (24)
Ethmoidal sinus (C31.1)	583 (24)	146 (16)
Frontal sinus (C31.2)	48 (2)	14 (2)
Sphenoidal sinus (C31.3)	113 (5)	51 (6)
Overlapping lesion of accessory sinuses (C31.8)	19 (1)	8 (1)
Accessory sinus, unspecified (31.9)	32 (1)	10 (1)
Histology ^(a)^		
Adenocarcinomas		
ITAC	678 (33)	74 (10)
Non-ITAC	93 (5)	42 (6)
AD NOS	160 (8)	26 (3)
SGC	112 (6)	108 (14)
Squamous cell carcinomas		
SCC	730 (36)	376 (50)
Neuroendocrine carcinomas		
NEC	43 (2)	21 (3)
Other epithelial neoplasms		
SNC NOS	108 (5)	49 (6)
SNUC	114 (6)	57 (8)
Malignant Neoplasm	16 (1)	9 (1)

^(a)^ AD NOS, adenocarcinoma not otherwise specified; ITAC, intestinal-type adenocarcinoma; NEC, neuroendocrine carcinoma; non-ITAC, non-intestinal-type adenocarcinoma; SCC, squamous cell carcinoma; SNC NOS, sinonasal carcinomas not otherwise specified; SGC, salivary gland carcinoma; SNUC, sinonasal undifferentiated carcinoma.

**Table 2:** Gender differences in SNC incidence rates (per 100,000 person-years) by age class, cancer site, and main histologic groups (period: 2015–2018).

Variable	Incidence Rate		IRR (95% CI)
	Men	Women	
Age-class			
0–24	-	-	-
25–34	0.01	0.01	0.98 (0.28–3.37)
35–44	0.04	0.03	1.22 (0.70–2.12)
45–54	0.09	0.03	2.83 (1.82–4.39)
55–64	0.15	0.04	3.55 (2.44–5.19)
65–74	0.22	0.06	3.42 (2.54–4.62)
75+	0.26	0.06	4.05 (3.11–5.27)
Topography (most represented)			
Nasal cavities (C30.0)	0.51	0.15	3.41 (2.82–4.13)
Maxillary sinus (C31.0)	0.16	0.06	2.47 (1.82–3.35)
Ethmoid sinus (C31.1)	0.18	0.04	4.01 (2.86–5.63)
Histology (main groups)			
Adenocarcinoma	0.35	0.07	5.00 (3.83–6.53)
Squamous cell carcinoma	0.30	0.13	2.31 (1.85–2.88)

Note: IRR: incidence rate ratio; CI: confidence interval.

## Data Availability

The data presented in this study are available in this article (and Supplementary Material).

## Supplementary Materials

The following supporting information can be downloaded at https://www.mdpi.com/article/10.3390/cancers16112053/s1, Figure S1: Spatial coverage of the ReNaTuNS surveillance system by region. Italy, 1993–2018; Figure S2: Forest plot of incidence rate ratios (IRRs) by Italian Region with 95% confidence intervals; Figure S3: Distribution of exposures by direct and indirect interview (period: 1989–2022); members of the ReNaM working group.
