# Supplementary material for: Gender Differences in Sinonasal Cancer Incidence: Data from the Italian Registry
Source: Cancers (Basel). 2024 May 29;16(11):2053. doi: 10.3390/cancers16112053 (PMC11171114; doi:10.3390/cancers16112053)
Supplement: Supplementary file 1 [file cancers-16-02053-s001.zip › cancers-3022137-supplementary.pdf]

# Gender Differences in Sinonasal Cancer Incidence: Data from the Italian Registry

**Members of the ReNaTuNS working group**: Alessandra Binazzi[1], Michela Bonafede[1], Davide di Marzio[1], Alessandro Marinaccio[1], Stefania Massari[1], Dario Consonni[2], Barbara Dallari[2], Carolina Mensi[2], Sabrina Rugarli[2], Simona Stella[2], Lucia Miligi[3], Valentina Cacciarini[4], Lucia Giovannetti[4], Paola Piccini[4], Sara Piro[4], Denise Sorasio[5], Jana Zajacovà[5], Angela Camagni[6], Paolo Galli[6], Massimiliano Marzadori[6], Roberto Calisti[7], Stefania Massacesi[7], Laura Ancona[8], Valeria Ascoli[8], Anna Balestri[8], Ilaria Cozzi[8], Tiziana Moiola[9], Stefano Murano[9], Lucia Rossin[9], Veronica Casotto[10], Vera Comiati[10], Ugo Fedeli[10], Manuel Zorzi[10], Silvia Eccher[11], Sara Lattanzio[11]

1 Department of Occupational and Environmental Medicine, Epidemiology, Hygiene, National Institute for Insurance against Accidents at Work (INAIL), 00100 Roma, Italy
2 Sinonasal Cancer Registry of Lombardy, Epidemiology Unit, Fondazione IRCCS Ca' Granda Ospedale Maggiore Policlinico, 20100 Milano, Italy
3 Institute for Cancer Research, Prevention and Clinical Network (ISPRO) Foundation and already Sinonasal Cancer Registry of Tuscany, Occupational and Environmental Epidemiology Unit,(ispro), 50139 Firenze, Italy
4 Sinonasal Cancer Registry of Tuscany, Occupational and Environmental Epidemiology Unit, (ISPRO), 50139 Firenze, Italy
5 Sinonasal Cancer Registry of Piedmont, Occupational Health and Safety Department, CN1 Local Health Authority, 12037 Saluzzo, Italy
6 Sinonasal Cancer Registry of Emilia Romagna, Occupational Safety and Prevention Unit, Public Health Department, Bologna Local Health Authority, 40121 Bologna, Italy
7 Sinonasal Cancer Registry of Marche, Unit of Workplace Prevention and Safety and of Occupational Epidemiology (SPreSAL Epi Occ), Department of Prevention, Macerata Health Authority, 62012 Civitanova Marche, Italy
8 Sinonasal Cancer Registry of Lazio, Department of Epidemiology, Lazio Regional Health Service, ASL Roma 1, 00147, Roma, Italy, and Department of Prevention -Laboratory of Industrial Hygiene-CRRA ASL Lazio 01100, Viterbo, Italy
9 Sinonasal Cancer Registry of Autonomous Province of Bolzano, Alto Adige Health Authority, Occupational Medicine Unit, 39100 Bolzano, Italy
10 Azienda Zero, Epidemiological Department, Veneto Region, 35131 Padova, Italy
11 Sinonasal Cancer Registry of Autonomous Province of Trento, Hygiene and Occupational Medicine, Provincial Unit of Health, 38123 Trento, Italy

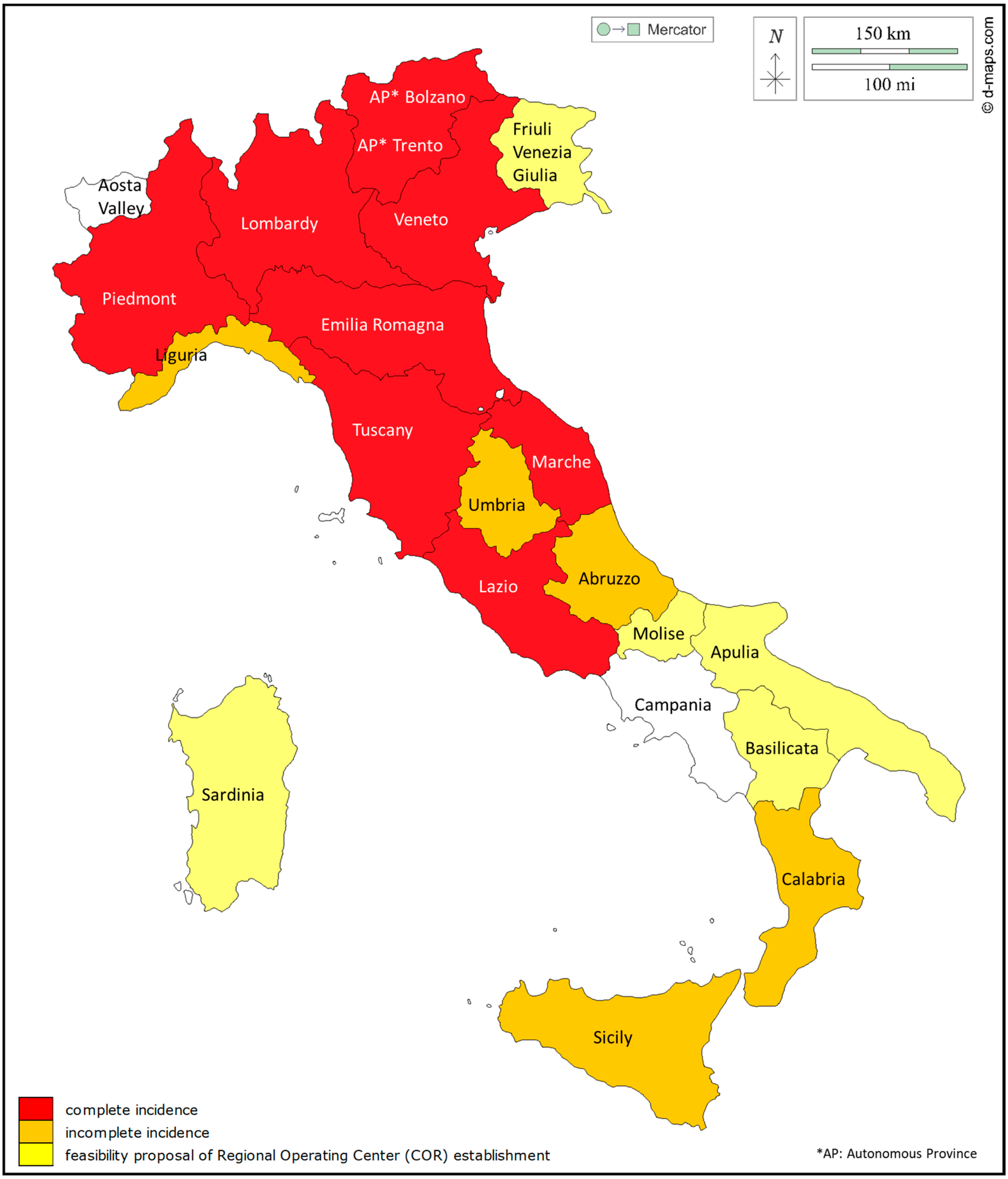


**Figure S1.** Spatial coverage of the ReNaTuNS surveillance system by Region. Italy, 1993–2018.

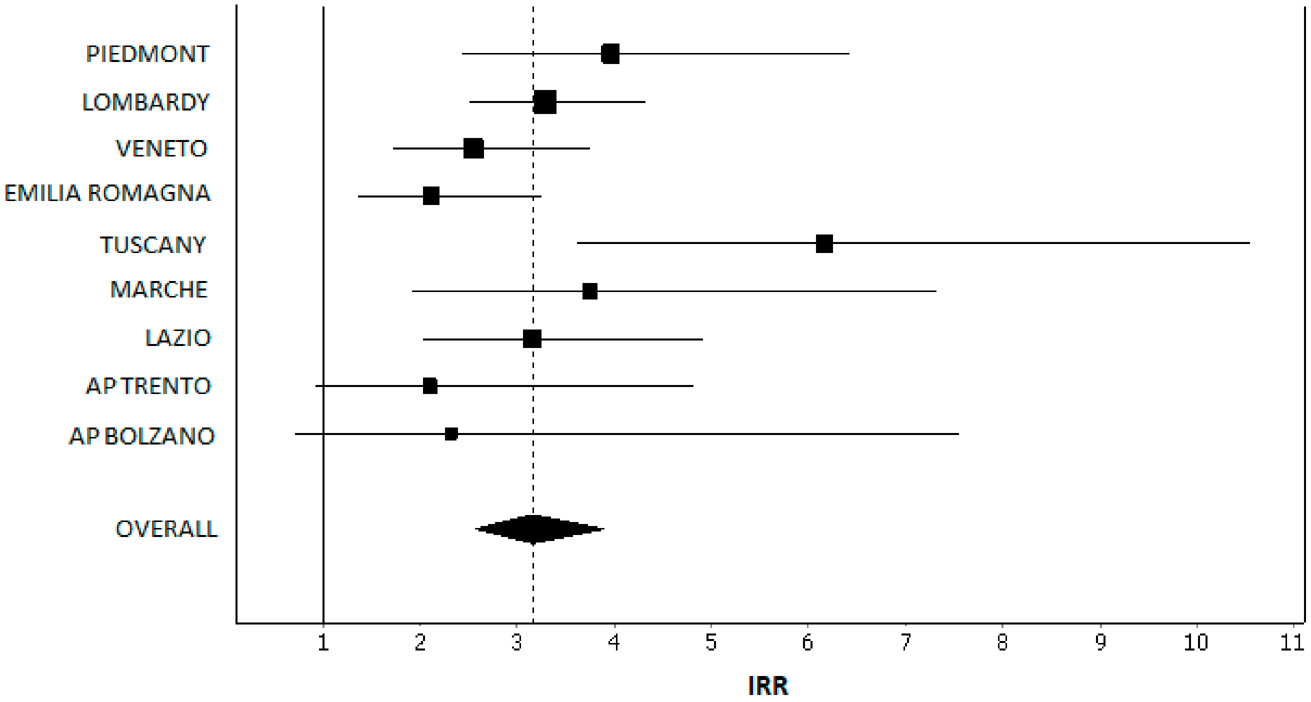


AP: Autonomous Province

**Figure S2**. Forest plot of Incidence Rate Ratios (IRRs) by Italian Region with 95% confidence intervals.

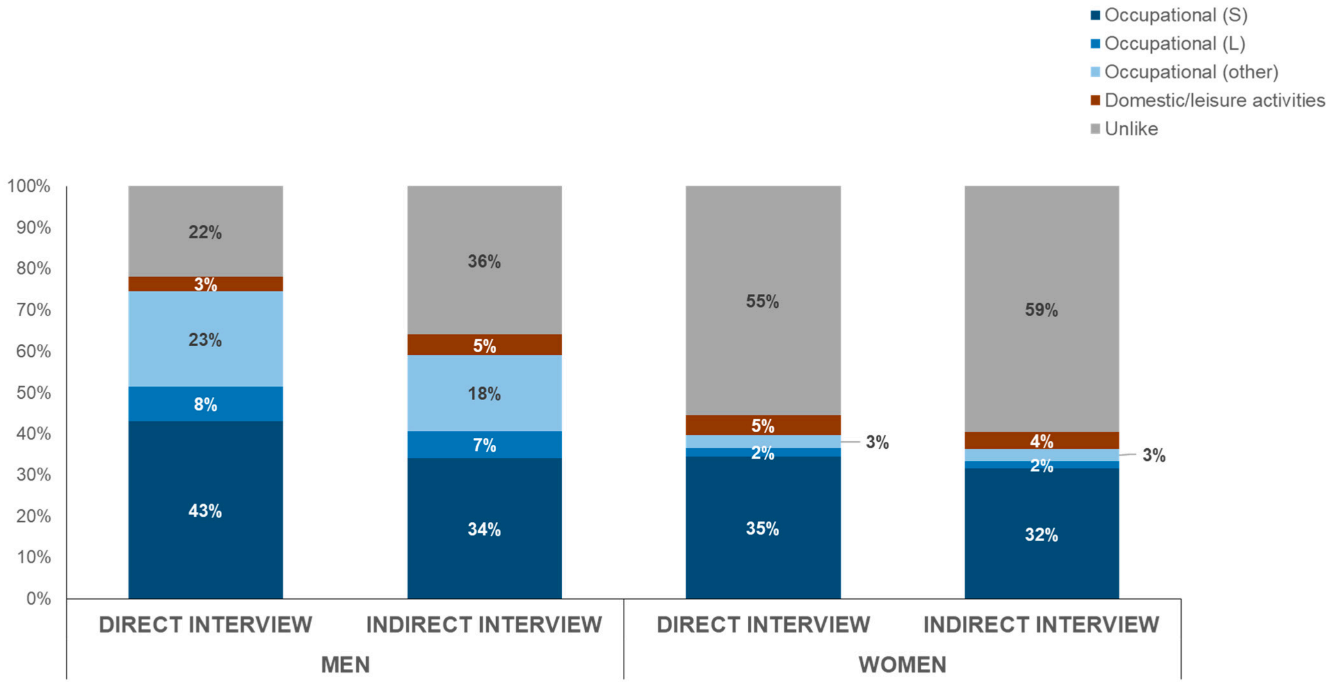


**Figure S3.** Distribution of exposures by direct and indirect interview (period: 1989–2022).

Note: S = carcinogenic agents with sufficient evidence in humans for SNCs; L = carcinogenic agents with limited evidence in humans for SNCs
